# Synergistic Effects of B-F/B-S and Nitrogen Vacancy Co-Doping on g-C_3_N_4_ and Photocatalytic CO_2_ Reduction Mechanisms: A DFT Study

**DOI:** 10.3390/molecules27217611

**Published:** 2022-11-06

**Authors:** Gang Fu, Xiaozhuo Song, Siwei Zhao, Jiaxu Zhang

**Affiliations:** School of Chemistry and Chemical Engineering, Harbin Institute of Technology, Harbin 150001, China

**Keywords:** g-C_3_N_4_ nonmetallic element co-doping, nitrogen vacancies, electronic structure modification, photocatalytic CO_2_ reduction mechanism

## Abstract

Nonmetallic co-doping and surface hole construction are simple and efficient strategies for improving the photocatalytic activity and regulating the electronic structure of g-C_3_N_4_. Here, the g-C_3_N_4_ catalysts with B-F or B-S co-doping combined with nitrogen vacancies (N_v_) are designed. Compared to the pristine g-C_3_N_4_, the direction of the excited electron orbit for the B-F-co-doped system is more matching (N_2pz_→C_2pz_), facilitating the separation of electrons and holes. Simultaneously, the introduced nitrogen vacancy can further reduce the bandgap by generating impurity states, thus improving the utilization rate of visible light. The doped S atoms can also narrow the bandgap of the B-S-N_v_-co-doped g-C_3_N_4_, which originates from the p-orbital hybridization between C, N, and S atoms, and the impurity states are generated by the introduction of N vacancies. The doping of B-F-N_v_ and B-S-N_v_ exhibits a better CO_2_ reduction activity with a reduced barrier for the rate-determining step of around 0.2 eV compared to g-C_3_N_4_. By changing F to S, the origin of the rate-determining step varies from *CO_2_→*COOH to *HCHO→*OCH_3_, which eventually leads to different products of CH_3_OH and CH_4_, respectively.

## 1. Introduction

Fossil fuels are a vital source of energy for human beings [1]; however, the use of fossil fuels releases CO_2_ to accumulate in the atmosphere, leading to a serious greenhouse effect [2,3]. Finding efficient ways to reduce CO_2_ emissions or capture CO_2_ and convert it into chemical commodities is the key problem to solve [4,5,6,7]. Lightweight materials with different nano-morphologies containing heteroatoms have been actively pursued for catalytic applications and as semiconductor materials because of their unusual properties [8]. Graphitic carbon nitride (g-C_3_N_4_) is a star two-dimensional metal-free semiconductor material with a bandgap of 2.7 eV [9]. Unfortunately, the catalytic reduction of CO_2_ using pristine g-C_3_N_4_ is very inefficient due to the fast recombination of photogenerated holes and electrons and the low utilization efficiency of visible light [10,11]. To improve the photocatalytic efficiency of pristine g-C_3_N_4_, many studies have been carried out, including structure optimization, doping modification [12,13,14,15], structural and defect engineering [16], and composite heterojunction materials [17,18,19,20,21,22,23]. Experimental studies have shown that nonmetallic doping, such as B, O, S, P, and halogen doping, is an effective way to enhance the photocatalytic performance and maintains the metal-free photocatalytic properties of g-C_3_N_4_ [24,25,26,27]. Yan found that boron-doped g-C_3_N_4_ effectively utilized visible light, increasing the photocatalytic degradation rate of rhodamine B by 3.6 times [8], while Ohno et al. applied it in the photocatalytic CO_2_ reduction process and generated methanol [24]. In the halogen-doped system, the F-doped g-C_3_N_4_ was reported to have the smallest bandgap energy and the strongest light absorption ability, and the doped g-C_3_N_4_ was corrugated to enhance the structural stability, but the adsorption energy of CO_2_ was small, which was not conducive to the subsequent restoration process [22]. Liu, and Wang et al. [6,28] reported the S-doped g-C_3_N_4_ material; the bandgap was significantly reduced, the adsorption of CO_2_ was enhanced, and it was reduced to methanol, but the reason for the separation of electrons and holes was not explained.

Recently, researchers have focused on the double-element doped system. The characteristics of continuous tuning can be exhibited by changes in the composition of host and guest elements and flexible modification of the electronic structure and/or geometry of the two-element catalysts during synthesis, which greatly improves the ability to enhance the catalytic activity, selectivity, and stability and reduce catalyst cost [29,30]. Cui. et al. studied the B-F system, proving that the potential of the conduction band was sufficient to reduce CO_2_, but the expansion of the bandgap limited the further development in the field of photocatalysis application [25]. Han et al. studied the B-S system and demonstrated the important synergistic effect of nonmetallic co-doping on the photocatalytic reduction of CO_2_ by increasing the utilization of visible light [26]. Tay, and Tu et al. proved that the introduction of the nitrogen vacancy (N_v_) further improved the performance of metal-co-doped materials [27,31]. Wang et al. further revealed in the B-K-N_v_ experiment that N_v_ can enrich electrons and was more prone to chemical reactions [32]. However, metals tend to form TM-H bonds with hydrogen during catalysis, leading to a low Faraday efficiency. In addition, metal ions tend to spill over and cause environmental pollution [33,34]. The focus of research has shifted to catalysts doped with nonmetallic elements, which, to some extent, eliminates the environmental problems of metallic catalysts [35]. Defect engineering is another effective strategy to modulate catalyst performance, which improves catalytic performance by introducing impurity states to reduce the bandgap [36]. In order to investigate how vacancies interact with nonmetallic elements to improve catalytic performance, this paper designs the nonpolluting and efficient catalysts using double nonmetallic elements coupled with a nitrogen vacancy to investigate the microscopic reaction mechanism of carbon dioxide reduction.

First-principles calculations within the Density Functional Theory (DFT) method have contributed to the understanding of modifications at the atomic scale [37]. Considering the advantages of B, F, and S dopants of the narrower bandgap, stronger photocurrent response [8], sufficient utilization of visible light [22], and promotion of the separation of photogenerated carriers [38,39], in this work, the nonmetal dual elements catalysts, e.g., the B-F-N_v_-co-doped g-C_3_N_4_ (abbreviated as B-F-N_v_) catalyst, as well as the B-S-N_v_-co-doped g-C_3_N_4_ (abbreviated as B-S-N_v_), were designed. The nitrogen vacancy was also introduced here, as it could introduce additional energy levels and/or act as reaction sites [40,41]. Importantly, the possible reaction pathways for CO_2_ reduction to C1 products were further investigated. It is of interest to investigate how the nonmetallic co-doping combined with nitrogen vacancies synergistically modulates the photocatalytic performance and facilitates reduction of CO_2_. By calculating the Gibbs free energy change (ΔG) and the product adsorption energy, the species of CO_2_ final reduction products were determined.

## 2. Computational Methods

The electronic structural properties of the pristine and doped g-C_3_N_4_ catalysts were calculated using the Vienna Ab-initio Simulation Package (VASP) [42], and the projected plus plane wave (PAW) pseudopotential [40] was used to describe the interaction between ionic real and valence electrons. The unit cell energy and lattice constant were calculated using the PBE functional of the generalized gradient approximation method (GGA) [43]. During the structural optimization calculations, a very precise accuracy of 1.0 × 10^−6^ eV was used as the criterion for energy convergence. For the K-point setting of the system, the Mokhorst–Pack setting of 3 × 3 × 1 was used in the structural relaxation, and the K-point setting of 6 × 6 × 1 was used in the static calculation and performance calculation. Considering that the PBE method tended to underestimate the bandgap value, the band structures of the pristine and doped systems were calculated using the HSE06 hybrid functional [44,45]. In order to avoid mutual interference between adjacent systems, a vacuum space of 15 Å was introduced and the energy cutoff value of the system was set to 450 eV, and the effect of spin polarization was considered in the calculation. The PBE (D3) method with the Grimme van der Waals correction was employed because of the weak interactions between CO_2_ Photocatalytic Reduction (CO_2_RR) Reaction species and catalysts [46].

**Figure 1:**
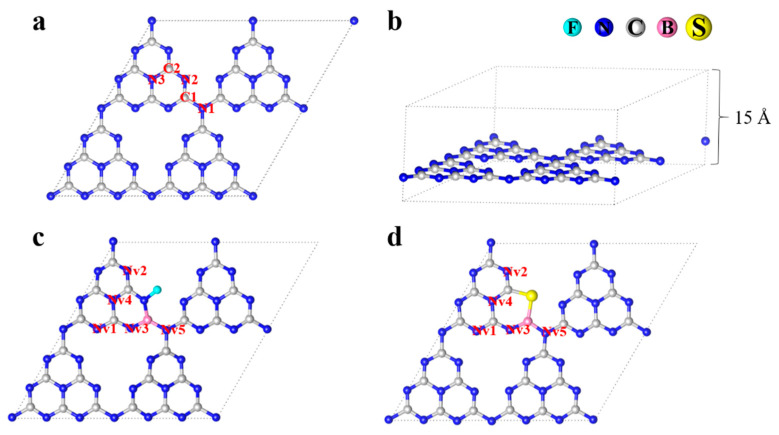
Pristine and doped g-C_3_N_4_ geometries. (**a**) Top view of pristine g-C_3_N_4_ geometry. (**b**) Side view of pristine g-C_3_N_4_ geometry. (**c**) N_v_ sites of B-F-N_v_. (**d**) N_v_ sites of B-S-N_v_.

Pure bulk g-C_3_N_4_ is built on the hexagonal heptadiazine-based structure as it is the most stable graphitic phase [47], and it contains two or three different kinds of C and N atoms, which are marked as C1-C2 and N1-N3, respectively, as shown in Figure 1a,b. According to the experimental results, B-N bonds and N-F bonds existed in the XPS spectra of B-F-co-doped g-C_3_N_4_ [25], indicating that B atoms preferentially replaced C atoms (C1), and F atoms tended to combine with N atoms (N2) to be doped into the interspace, as shown in Figure 1c. There are 5 possible sites where N_v_ can be introduced, namely, N_v_1-N_v_5 in B-F-co-doped g-C_3_N_4_, as shown in Figure 1c. For B-S-co-doped g-C_3_N_4_, similar possible doping sites are provided, as given in Figure 1d, based on the experiment [26]. The optimal doping site can be determined by the formation energy (E_form_), according to the following equation:

E_form_ = E_doped_ + μB − E_undoped_ − μA (1)

E_undoped_ and E_doped_ are the electron energies of the catalysts before and after doping, respectively. μA and μB are the chemical potentials of the substituted atoms, i.e., μC, μN, μB, μF, and μS. Formation energy can also reflect the difficulty of doping; the lower the formation energy, the better the thermodynamic stability.

## 3. Results and Discussion

### 3.1. Geometric Structure and Thermodynamic Stability

The formation energy results are shown in Table S1 to determine the location of nitrogen vacancies. The E_form_ of the doped N_v_4 site of B-F-N_v_ is lowest among the possible doping sites, indicating that it is the most stable structure, and the negative value of −0.55 eV suggests that the introduction of N_v_ is thermodynamically spontaneous [41] and easier to synthesize experimentally. In contrast, the lowest E_form_ of B-S-N_v_ is 1.07 eV, corresponding to the doped site of N_v_2, suggesting that it is more difficult to introduce N_v_ on the basis of B-S than B-F-doped g-C_3_N_4_.

**Figure 2:**
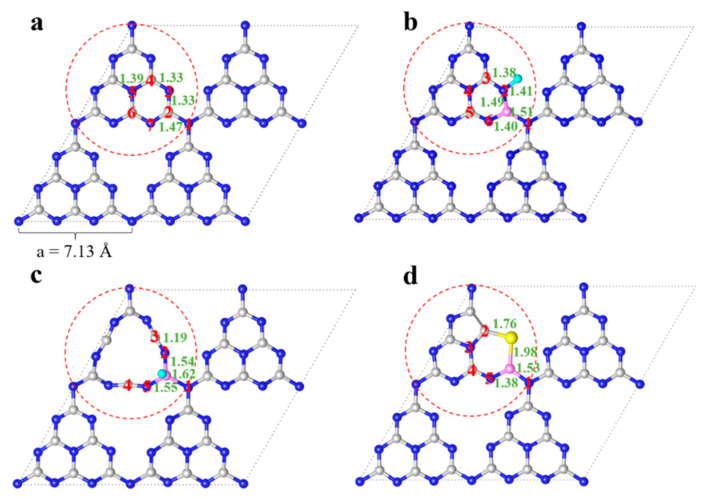
The optimized geometries and bond lengths of g-C_3_N_4_ (**a**), B-F (**b**), B-F-N_v_ (**c**), and B-S-N_v_ (**d**).

To explain how the doped elements and vacancies affect the properties of pristine g-C_3_N_4_, the optimized bond length parameters for pure g-C_3_N_4_, B-F, and B-F-N_v_-doped g-C_3_N_4_ are shown in Figure 2, Table S2 and compared with the experimental [48] and theoretical values [28]. It can be seen that the optimized geometric parameters agree well with the available results; the maximum deviations from theory and experiment are 0.02 and 0.13 Å, respectively, verifying the reliability of the calculation method and parameter settings. Figure 2a–d show the variation in the key bond lengths from g-C_3_N_4_, B-F, and B-F-N_v_ to B-S-N_v_. Compared to the pristine g-C_3_N_4_, the introduction of elements and holes only changes the local geometry of the doping site and has little effect on other sites for the doped g-C_3_N_4_. The dramatic change lies in B (C2)-N1 bonds, which is elongated from 1.47 and 1.51 to 1.62 Å gradually from g-C_3_N_4_ to the introduction of B and F atoms and then the nitrogen vacancies. This is because the radius of the B atom (0.88) is larger than that of the C atom (0.77) and the electronegativity of the F atom (3.98) is stronger. For B-F-co-doped g-C_3_N_4_, the introduction of the F atom with the stronger electronegativity can facilitate the electrons flowing to the F atom, thus weakening the B-N bond. With the further introduction of N_v_, the F atom shifts from N2 to the B atom, and importantly, the C atom changes from sp^2^ hybridization to sp hybridization, leading to the enhanced electronegativity of the C atom. Thus, the interaction between the C and the N atom is further enhanced, and the interaction between the N and the B atom is weakened, leading to the further elongation of the B-N bond. Similarly, for B-S-N_v_, the introduction of B and S elements and N_v_ extends the distance of the B-N1 bond to 1.53 Å. Due to the absence of N, the structure changes from a six-membered ring to a five-membered ring at the nitrogen vacancy ring, and local large deformation occurs. Generally, with the degree of local deformation of the geometric structure increased, the mutual repulsion between the lone pairs of electrons of the N atom in the system will reduce and the material will become more stable [49].

### 3.2. Electronic Structure and Electronic Properties

The bandgaps of g-C_3_N_4_, and B-F-, B-F-N_v_-, and B-S-N_v_-co-doped g-C_3_N_4_ calculated by different methods are listed in Table S3 together with experimental and literature values [8,14,25]. The bandgap values calculated by the HSE06 hybrid functional in this paper are in good agreement with those of previous studies [24,50], again testifying the rationality of the structures. Due to the limitations of the GGA method, the multi-electron interactions cannot be fully described, and the bandgap value is often underestimated, but it does not affect the results of qualitative comparisons and the rules. The results show that B-F co-doping increases the bandgap value from 2.77 to 3.06 eV compared to g-C_3_N_4_, while the injection of N_v_ significantly reduces the bandgap value from 3.06 to 2.67 eV, which facilitates the broadening of the visible light utilization.

**Figure 3:**
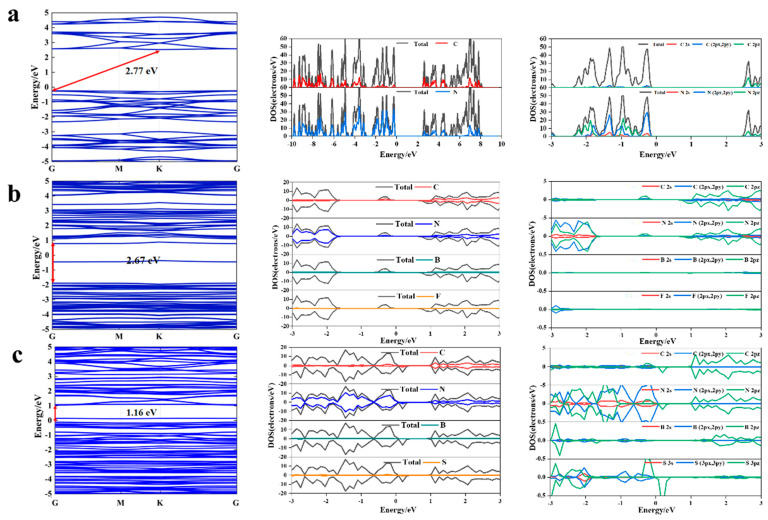
Calculated band structure (**left**), density of states (**middle**), and partial density of states (**right**) diagrams of g-C_3_N_4_ (**a**), B-F-N_v_ (**b**), and B-S-N_v_ (**c**).

The band structure and contribution of atoms and atomic orbitals to the density of states for g-C_3_N_4_, and B-F-N_v_- and B-S-N_v_-co-doped g-C_3_N_4_ are presented in Figure 3. It can be seen that the valence band maximum (VBM) and conduction band minimum (CBM) for g-C_3_N_4_ are located at point Gamma and point K, respectively. Due to the different positions of the VBM and CBM in k-space, g-C_3_N_4_ is an indirect bandgap semiconductor with a bandgap value of 2.77 eV calculated by the HSE06 method. The valence band is mainly contributed by N_(2px, 2py)_ orbitals of N atoms, while the conduction band is mainly C_2pz_ of C atoms and a small amount of N_2pz_ orbitals of N atoms. Due to the different orbital orientations, it is difficult to excite electrons from N_(2px, 2py)_ to C_2pz_ or N_2pz_ orbitals, which is one of the reasons for the difficult transition of photogenerated electrons and low carrier mobility in g-C_3_N_4_ [51].

In contrast, B-F-N_v_ is a direct bandgap structure with a value of 2.67 eV calculated by the HSE06 method, smaller than g-C_3_N_4_, as both VBM and CBM are located at point Gamma. The direct transition of electrons reduces the energy loss caused by electron relaxation, which means a higher energy conversion efficiency [52,53,54]. The loss of nitrogen atoms caused by vacancies destroys the π-conjugation of g-C_3_N_4_, leading to the reconstruction of the band structure and the formation of impurity states in the middle of the forbidden band. Due to the low doping concentration of B (1.73 at.%) and F (1.75 at.%) atoms, the contribution of both types of atoms to the band edges is not obvious [49]. The introduction of nitrogen vacancies generates additional electron and spin polarization in g-C_3_N_4_, and an impurity state is created in the middle of the forbidden band of the spin states. The valence band consists mainly of N_2pz_ and N_(2px, 2py)_ orbitals and the conductive band is mainly composed of C_2pz_ orbitals. As the electron transition orbital direction is the same, it is easier to realize the electron transition and improve the carrier mobility. The impurity state consists of C_2pz_ and N_2pz_ orbitals, which belong to the hanging bonds of carbon atoms in nitrogen vacancies [27]. The composition of the impurity state is consistent with the composition at the bottom of the conduction band, which can receive the electrons excited at the top of the valence band and shift the light absorption wavelength to the long-wave direction. This facilitates continuous photoexcitation of the VBM to CBM in the visible light range.

Compared to g-C_3_N_4_ and B-F-N_v_, B-S-N_v_ is also a direct bandgap structure and the doped S atoms can further narrow the bandgap, as shown in Table S3. The PBE method is used to calculate the bandgap values for g-C_3_N_4_, B-F-N_v_, and B-S-N_v_ as 1.21, 1.19, and 1.16 eV, respectively. By contrast, the impurity level is generated at near the VBM due to the introduction of nitrogen vacancies, which is mainly contributed by the N_2pz_ orbital. As shown in Figure 3c, the VBM is contributed by N atoms and a small amount of C and S atoms. The overlap of electronic states of S and C indicates the hybridization of S and C atomic orbitals, while the CBM is mainly contributed by C atoms and a small amount of N atoms. The B atoms have little contribution to the energy band edges and do not directly participate in the generation, separation, and migration of photogenerated carriers, but act as electron donors to enhance the photocatalytic performance of g-C_3_N_4_ by affecting the charge distribution of surrounding atoms [8]. The formed impurity state at the top of the valence band of the spin-down state consists of hybridized C_2pz_, N_2pz_, and S_3pz_ orbitals. This is because the doped S atoms affect the distribution of C and N atoms in the lattice, resulting in p-orbital hybridization between C, N, and S atoms.

**Figure 4:**
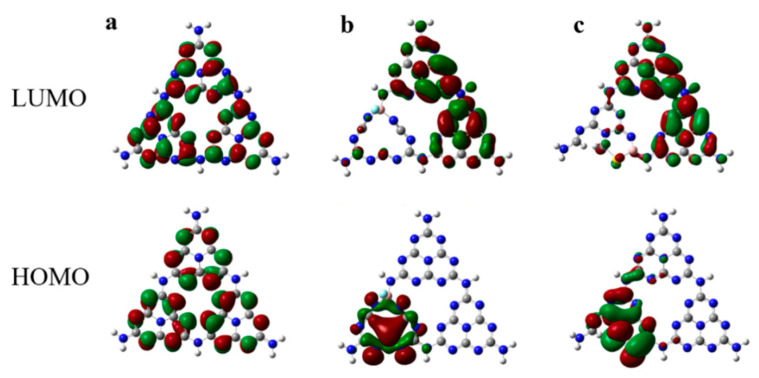
HOMO and LUMO for g-C_3_N_4_ (**a**), B-F-N_v_ (**b**), and B-S-N_v_ (**c**).

Figure 4 presents the highest occupied molecular orbital (HOMO) and the lowest unoccupied molecular orbital (LUMO) of g-C_3_N_4_, B-F-N_v_, and B-S-N_v_. For g-C_3_N_4_, the HOMO covers all the corner N atoms, while the LUMO is mainly distributed on the C and N atoms. The bridge N atoms are not involved in either the valence or the conduction band, so they do not excite or accept electrons, hindering the transfer of charge carriers between the heptylhydrazine rings by bridging N atoms. Both photogenerated electrons and holes are concentrated on each heptylhydrazine ring, resulting in a high carrier recombination rate and poor photocatalytic activity due to the spatial overlap of HOMO and LUMO orbitals. In contrast, the charge density rearranges and electron-rich regions appear for both B-F-N_v_ and B-S-N_v_. The HOMO orbitals are mainly distributed on the N_v_-introduced building blocks, while the LUMO orbitals are mainly on the remaining unmodified heptyltriazine rings, which differs from the spatial overlap of the HOMO and LUMO orbitals of g-C_3_N_4_, as shown in Figure 4b,c. The HOMO and LUMO orbitals of B-F-N_v_ and B-S-N_v_ are completely spatially separated, which will effectively improve the separation efficiency of photogenerated electrons and holes. In addition, the HOMO and LUMO orbitals are also distributed on the bridged N atoms, which facilitates the carrier migration between the heptatriazine ring building blocks and increases the carrier mobility [55,56].

### 3.3. Adsorption Properties of CO_2_

The effects of modification methods on the electronic and optical properties of g-C_3_N_4_ have been focused on extensively; however, the theoretical studies of their applications on specific photocatalytic reactions are still limited. In this work, the effects of B-F-N_v_ and B-S-N_v_ modification on the photocatalytic CO_2_ reduction of g-C_3_N_4_ are investigated. The first step of the photocatalytic reaction is the adsorption of CO_2_. Figure S1 presents the possible adsorption sites for CO_2_ on B-F-N_v_ and B-S-N_v_, together with adsorption energy, to determine the optimal adsorption site. The adsorption energy of CO_2_ placed above the nitrogen vacancy in parallel (Figure S1a,b) and vertically (Figure S1c) is −0.16 eV, while that placed above the interstitial in parallel (Figure S1d) is −0.26 eV, indicating that the optimal adsorption site is above the interstitial. This may be because after modification and under the visible light excitation, holes appear around the nitrogen vacancies to generate oxidation sites and exhibit oxidation activity, while the charge density increases from −1.12 e to −1.39 e on the corner N atoms around the interstitial, as shown in Figure S3 (red cycle). Therefore, the optimal adsorption site of CO_2_ on B-F-N_v_ is not the nitrogen vacancy with strong oxidation activity, but the interstitial with strong reduction activity.

**Figure 5:**
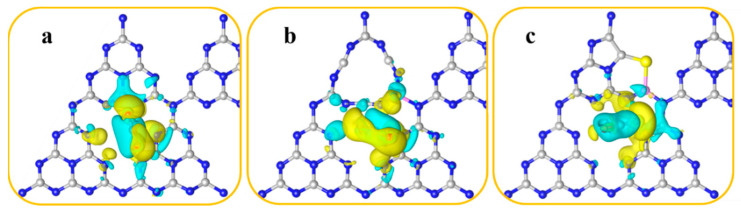
The map of differential charge density for g-C_3_N_4_ (**a**), B-F-N_v_ (**b**), and B-S-N_v_ (**c**).

For B-S-N_v_, the adsorption energy is −1.99, −2.68, and −1.97 eV for CO_2_ adsorbed above the nitrogen vacancies, above the interstitial, and on top of B and S atoms, respectively, as shown in Figure S1e–g. Thus, the optimal adsorption site of CO_2_ on B-S-N_v_ is also above the interstitial, which is similar to B-F-N_v_, but the adsorption of CO_2_ on B-S-N_v_ is stronger than on B-F-N_v_. This can be explained by the charge transfer determined by the differential charge density (CDD) map. Figure 5 is a CDD plot of CO_2_ adsorption on g-C_3_N_4_, B-F-N_v_, and B-S-N_v_. The yellow and blue color represents electron enrichment and depletion, respectively, so charge flows from the blue area to the yellow area. In the g-C_3_N_4_ adsorption system, there are less charge distributions on the interstitial edge corner N atoms and bridging N atoms. In the B-F-N_v_ adsorption system, the charge transfers from the B atom to the adjacent N atom active site, and the charge around the nitrogen vacancy flows to the adsorption active site, forming a charge-rich environment at the edge N atom site. F atoms serve as bridges to transfer electrons to CO_2_. However, the charge density on the bridging F atom is too high, and the electron repulsion between the bridging F and the O atom of CO_2_ becomes larger, weakening the adsorption effect. In contrast, for the B-S-N_v_ adsorption system, the charge concentration flows to the adsorption active site, and the charge density enrichment degree on the corner N atoms at the gap edge increases, thereby enhancing the adsorption of CO_2_.

### 3.4. Catalytic Performance for the Photocatalytic CO_2_ Reduction

**Figure 6:**
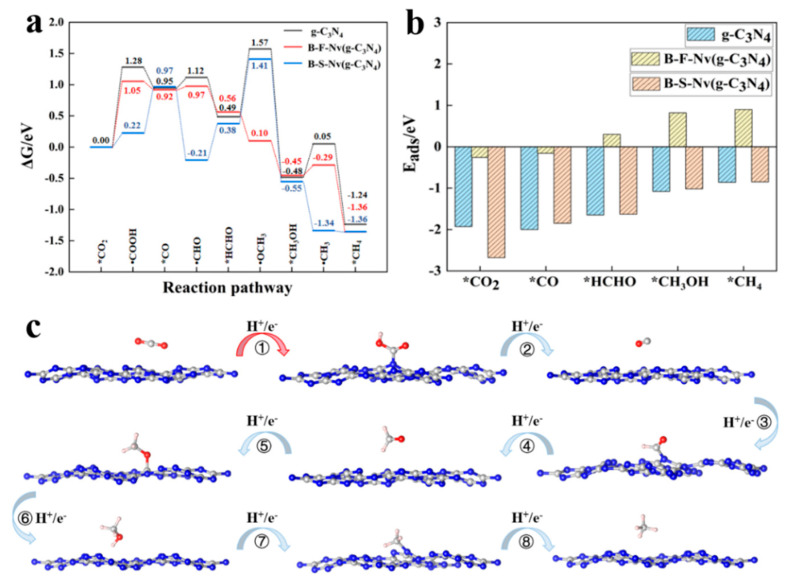
(**a**) Gibbs free energy diagrams of photocatalytic CO_2_ reduction and (**b**) adsorption ene-gies of products in different stages of CO_2_ reduction for pristine and doped systems. (**c**) The repr-sentative geometries of the stable points in the specific reduction process for CO_2_ on g-C_3_N_4_.

Carbon dioxide is a very stable linear molecule, and the photocatalysts with a suitable conduction band potential to convert carbon dioxide into hydrocarbon fuel are highly desirable. The selectivity of g-C_3_N_4_ to the reduction product is still controversial and the yield is low. In addition, how the doping modification affects the catalytic performance and product selectivity of g-C_3_N_4_ should also be investigated. The Gibbs free energy curve and adsorption energies of products in different stages for g-C_3_N_4_, B-F-N_v_, and B-S-N_v_ are presented in Figure 6, as well as the representative geometries of the stable points in the specific reduction process of CO_2_ on g-C_3_N_4_. The photocatalytic CO_2_ reduction pathways are similar for all three catalysts, i.e., *CO_2_→*COOH→*CO→*CHO→*HCHO→*OCH_3_→*CH_3_OH→*CH_3_→*CH_4_ as proposed by Azofra et al. [49]. The speculated probable products are CO, HCHO, CH_3_OH, and CH_4_, which are 2, 4, 6, and 8 electron processes, respectively. Further, Yu et al. [57]. Experimentally synthesized a g-C_3_N_4_ catalyst and applied it to the reduction of CO_2_ under UV-Vis radiation, confirming that the main product of CO_2_ reduction was CH_3_OH and that it reached a yield of 0.81 μmol·g^−1^. In the Gibbs free energy diagram calculated in this paper, it can be seen that △G = −0.48 eV when CO_2_ is reduced to CH_3_OH, and 0.53 eV of energy needs to be absorbed in the subsequent reaction. Therefore, it can be seen that methanol exists stably, and methanol is the final reduction product, which is consistent with the experiment [57].

For B-F-N_v_, the formation of free radicals is endothermic because breaking carbon-oxygen single or double bonds requires external input energy, i.e., *CO_2_→*COOH ①, *CO→*CHO ③, and *CH_3_OH→*CH_3_ ⑦, which is similar to g-C_3_N_4_. However, the *HCHO→*OCH_3_ ⑤ process for B-F-N_v_ releases 0.46 eV of energy, which is contrary to g-C_3_N_4_. As shown in Figure S3 (blue cycle), the charge on the adsorption site of the C atom for B-F-N_v_ is +1.60, which is larger than those of g-C_3_N_4_ and B-S-N_v_ with the values of +1.45 and +1.49, respectively. The increased charge density facilitates the formation of OCH_3_ to become an exothermic process, and thus is conducive to the subsequent reactions. The first hydrogenation process for B-F-N_v_ is the decisive step of the whole reduction reaction with the barrier of 1.05 eV, which is lower than that of g-C_3_N_4_ (1.28 eV), as shown in Figure 6a, facilitating the formation of COOH free radicals. In addition, the reaction releases energy (ΔG < 0) when a neutral product is formed, such as *·COOH→*CO ②, *CHO→*HCHO ④, *·OCH_3_→*CH_3_OH ⑥, *·CH_3_→*CH_4_ ⑧, and there is no chemical bond with the substrate in product of stage. However, the formed radicals such as ·COOH, ·CHO, ·OCH_3_, and ·CH_3_ can be bonded with the catalyst, and the adsorption sites are obviously different. For the species of C-terminal free radicals, they are easier to bond with the negatively charged N atom, and the species of O-terminated radicals are more likely to form chemical bonds with C atoms in the substrate, as shown in Figure 6c, which affects the elementary process of the reduction reaction to some extent. The energy of 0.55 eV is released when ·OCH_3_ transforms to CH_3_OH ⑥, and the adsorption energy is also favorable for desorption of methanol with the value of 0.82 eV, as shown in Figure 6b, so the final product is also CH_3_OH, which is similar to g-C_3_N_4_ and consistent with the previous experimental results [57].

Unlike B-F-N_v_, in the process of CO_2_ reduction on B-S-N_v_, there are two consecutive energy absorption processes ① and ②. The reaction barrier of the first hydrogenation with the value of 0.22 eV is much lower than those of both g-C_3_N_4_ and B-F-N_v_, which is favorable for the initial reaction. The reduction of HCHO to CH_3_O ⑤ is the rate-determining step by the absorption energy of 1.03 eV, lower than that of g-C_3_N_4_. Once crossing the reaction rate-determining step, it undergoes three exothermic processes until reduction to the final product CH_4_ with the small adsorption energy.

## 4. Conclusions

In this paper, the photocatalytic performance and photocatalytic CO_2_ reduction of the nonmetallic co-doping combined with nitrogen vacancy systems, B-F-N_v_ and B-S-N_v_, are investigated and compared to g-C_3_N_4_ to explore the doping effects. It is found that B-F-N_v_ and B-S-N_v_ can combine the advantages of the three modification methods to exert a synergistic effect. B-doping can compensate for the defect of the conduction band drop caused by F-doping and N_v_, and maintain the high reduction potential of CO_2_. With the doped F, B-F-N_v_ has a corrugated configuration, which reduces the repulsion between the lone pair electrons of the N atom, and improves the stability of the g-C_3_N_4_ structure. At the same time, F can act as a bridge for the transfer of charges from the doping system to CO_2_. N_v_ and B atoms form a charge-rich environment at the reactive site, which makes up for the charge loss caused by F doping and S doping.

The B-F-N_v_- and B-S-N_v_-co-doped g-C_3_N_4_ have a direct bandgap structure compared to the indirect one for g-C_3_N_4_. The introduction of heteroatoms and nitrogen vacancies affects the charge distribution and improves the separation of the HOMO and LUMO, thereby enhancing the separation efficiency of photogenerated electrons and holes. The synergistic effect of B-S-N_v_ makes the charge flow to the adsorption active site, which increases the charge enrichment of corner N atoms at the gap edge in g-C_3_N_4_, resulting in enhanced CO_2_ adsorption. B-F-N_v_- and B-S-N_v_-co-doped g-C_3_N_4_ have a lower rate-determining step than g-C_3_N_4_ for the rate-determining step photocatalytic CO_2_ reduction. B-S-N_v_ has the strongest adsorption capacity for CO_2_, and the desorption capacity of the reduction products is stronger than that of g-C_3_N_4_, but weaker than that of B-F-N_v_. The suggested main reduction product is CH_3_OH for CO_2_ reduction catalyzed by g-C_3_N_4_ and B-F-N_v_, and is CH_4_ catalyzed by B-S-N_v_. Therefore, the nonmetallic co-doping combined with N_v_ in this work provides a new idea for improving the catalytic performance of photocatalysts.

## Data Availability

The data presented in this study are available in supplementary material.

## Supplementary Materials

The following supporting information can be downloaded at: https://www.mdpi.com/article/10.3390/molecules27217611/s1, Figure S1: The possible adsorption sites for CO_2_ on B-F-N_v_ (a–d) and B-S-N_v_ (e–g), and corresponding adsorption energy (eV); Figure S2: The reaction pathways for B-F-N_v_ (a) and B-S-N_v_ (b); Figure S3: The bader charge (unit e) of g-C_3_N_4_ (left), B-F-N_v_ (middle), and B-S-N_v_ (right). Negative and positive values mean gaining and losing electrons. The elements are colored in pink for B, gray for C, blue for N, red for O, cyan for F, and yellow for S; Figure S4: Calculated band structure (left), density of states (middle), and partial density of states (right) diagrams of g-C_3_N_4_ (a), B-F-N_v_ (b) and B-S-N_v_ (c), the energy range is from −10 eV to 10 eV; Table S1: Formation energy (eV) of B-F-N_v_ and B-S-N_v_ co-doped g-C_3_N_4_; Table S2: The bond length parameters of intrinsic and doped g-C_3_N_4_ [28,48]; Table S3: The band gap energy (eV) for the intrinsic and doped g-C_3_N_4_ [28,31,50].

## Sample Availability

Samples of the compounds are not available from the authors.

## References

[1] Usubharatana P., McMartin D., Veawab A., Tontiwachwuthikul P. (2006). Photocatalytic Process for CO_2_ Emission Reduction from Industrial Flue Gas Streams. Ind. Eng. Chem. Res..

[2] Jing L., Zhou W., Tian G., Fu H. (2013). Surface Tuning for Oxide-Based Nanomaterials as Efficient Photocatalysts. Chem. Soc. Rev..

[3] Nakamura H. (2010). Recent Organic Pollution and its Biosensing Methods. Anal. Methods.

[4] Arumugam M., Tahir M., Praserthdam P. (2022). Effect of Nonmetals (B, O, P, and S) Doped with Porous g-C_3_N_4_ for Improved Electron Transfer towards Photocatalytic CO_2_ Reduction with Water into CH_4_. Chemosphere.

[5] Sun M., Zhao B., Chen F., Liu C., Lu S., Yu Y., Zhang B. (2021). Thermally-Assisted Photocatalytic CO_2_ Reduction to Fuels. Chem. Eng. J..

[6] Wang M., Li M., Liu Y., Zhang C., Pan Y. (2022). Structural Regulation of Single-Atomic Site Catalysts for Enhanced Electrocatalytic CO_2_ Reduction. Nano Res..

[7] Kemp K.C., Seema H., Saleh M., Le N.H., Mahesh K., Chandra V., Kim K.S. (2013). Environmental Applications using Graphene Composites: Water Remediation and Gas Adsorption. Nanoscale.

[8] Wang Y., Zhang J., Wang X., Antonietti M., Li H. (2010). Boron- and Fluorine-Containing Mesoporous Carbon Nitride Polymers: Metal-Free Catalysts for Cyclohexane Oxidation. Angew. Chem. Int. Ed..

[9] Wang X., Maeda K., Thomas A., Takanabe K., Xin G., Carlsson J.M., Domen K., Antonietti M. (2010). A Metal-Free Polymeric Photocatalyst for Hydrogen Production from Water under Visible Light. Nat. Mater..

[10] Wang Q., Fang Z., Zhang W., Zhang D. (2022). High-Efficiency g-C_3_N_4_ Based Photocatalysts for CO_2_ Reduction: Modification Methods. Adv. Fiber Mater..

[11] Wang S., Zhan J., Chen K., Ali A., Zeng L., Zhao H., Hu W., Zhu L., Xu X. (2020). Potassium-Doped g-C_3_N_4_ Achieving Efficient Visible-Light-Driven CO_2_ Reduction. ACS Sustain. Chem. Eng..

[12] Wang J., Song Y., Zuo C., Li R., Zhou Y., Zhang Y., Wu B. (2022). Few-Layer Porous Carbon Nitride Anchoring Co and Ni with Charge Transfer Mechanism for Photocatalytic CO_2_ Reduction. J. Colloid Interface Sci..

[13] Minhas N., Mustafa G., Kaur K., Kaur N., Singh G., Kaura A., Goswamy J.K. (2022). Alkali Metal Doping in B–C_3_N_4_ Extends Carrier Lifetime and Increases the CO_2_ Adsorption: DFT Study and Time-Domain Ab Initio Analysis. J. Phys. Chem. Solids.

[14] Zhu Y., Gong L., Zhang D., Wang X., Zhang J., Zhang L., Dai L., Xia Z. (2019). Catalytic Origin and Universal Descriptors of Heteroatom-Doped Photocatalysts for Solar Fuel Production. Nano Energy.

[15] Yang J., Jing L., Zhu X., Zhang W., Deng J., She Y., Nie K., Wei Y., Li H., Xu H. (2022). Modulating Electronic Structure of Lattice O-Modified Orange Polymeric Carbon Nitrogen to Promote Photocatalytic CO_2_ Conversion. Appl. Catal. B-Environ..

[16] Hou J., Yang M., Dou Q., Chen Q., Wang X., Hu C., Paul R. (2022). Defect Engineering in Polymeric Carbon Nitride with Accordion Structure for Efficient Photocatalytic CO_2_ Reduction and H_2_ Production. Chem. Eng. J..

[17] Li J., Li K., Du J., Yang H., Song C., Guo X. (2022). Impact of Transition Metal Incorporation on the Photocatalytic CO_2_ Reduction Activity of Polymeric Carbon Nitride. J. CO_2_ Util..

[18] Cao H., Yan Y., Wang Y., Chen F.-F., Yu Y. (2022). Dual Role of g-C_3_N_4_ Microtubes in Enhancing Photocatalytic CO_2_ Reduction of Co_3_O_4_ Nanoparticles. Carbon.

[19] Wu S., Mu Z., Fu G., Zhang J., Wang Y. (2022). Multi-Regulation of Charge Separation and Band Structure by a Novel O-doped g-C_3_N_4_ Nanosheets Homojunction for Enhanced Photodegradation Performance. J. Alloys Compd..

[20] Liu Q., Shen J., Yu X., Yang X., Liu W., Yang J., Tang H., Xu H., Li H., Li Y. (2019). Unveiling the Origin of Boosted Photocatalytic Hydrogen Evolution in Simultaneously (S, P, O)-Codoped and Exfoliated Ultrathin g-C_3_N_4_ Nanosheets. Appl. Catal. B-Environ..

[21] Wang Y., Di Y., Antonietti M., Li H., Chen X., Wang X. (2010). Excellent Visible-Light Photocatalysis of Fluorinated Polymeric Carbon Nitride Solids. Chem. Mater..

[22] Zhu B., Zhang J., Jiang C., Cheng B., Yu J. (2017). First Principle Investigation of Halogen-Doped Monolayer g-C_3_N_4_ Photocatalyst. Appl. Catal. B-Environ..

[23] Fu J., Liu K., Jiang K., Li H., An P., Wang X., Qiu X., Liu M. (2019). Graphitic Carbon Nitride with Dopant Induced Charge Localization for Enhanced Photoreduction of CO_2_ to CH_4_. Adv. Sci..

[24] Sagaraa N., Kamimura S., Tsubota T., Ohno T. (2016). Photoelectrochemical CO_2_ Reduction by a P-Type Boron-Doped g-C_3_N_4_ Electrode under Visible Light. Appl. Catal. B-Environ..

[25] Cui Y., Wang H., Yang C., Li M., Zhao Y., Chen F. (2018). Post-activation of in situ B-F Codoped g-C_3_N_4_ for Enhanced Photocatalytic H_2_ Evolution. Appl. Surf. Sci..

[26] Han X., Yao C., Yuan A., Xi F., Dong X., Liu J. (2018). Enhanced Charge Separation Ability and Visible Light Photocatalytic Performance of Graphitic Carbon Nitride by Binary S, B Co-Doping. Mater. Res. Bull..

[27] Tay Q., Kanhere P., Ng C.F., Chen S., Chakraborty S., Huan A.C.H., Sum T.C., Ahuja R., Chen Z. (2015). Defect Engineered g-C_3_N_4_ for Efficient Visible Light Photocatalytic Hydrogen Production. Chem. Mater..

[28] Wang Y., Tian Y., Yan L., Su Z. (2018). DFT Study on Sulfur-Doped g-C_3_N_4_ Nanosheets as a Photocatalyst for CO_2_ Reduction Reaction. J. Phys. Chem. C.

[29] Zhang J., Gao Z., Wang S., Wang G., Gao X., Zhang B., Xing S., Zhao S., Qin Y. (2019). Origin of Synergistic Effects in Bicomponent Cobalt Oxide-Platinum Catalysts for Selective Hydrogenation Reaction. Nat. Commun..

[30] Tao F.F. (2012). Synthesis, Catalysis, Surface Chemistry and Structure of Bimetallic Nanocatalysts. Chem. Soc. Rev..

[31] Tu W.-G., Xu Y., Wang J., Zhang B., Robertson J., Kraft M., Xu R. (2017). Investigating the Role of Tunable Nitrogen Vacancies in Graphitic Carbon Nitride Nanosheets for Efficient Visible-Light-Driven H_2_ Evolution and CO_2_ Reduction. ACS Sustain. Chem. Eng..

[32] Wang K., Fu J., Zheng Y. (2019). Insights into Photocatalytic CO_2_ Reduction on C_3_N_4_: Strategy of Simultaneous B, K Co-Doping and Enhancement by N Vacancies. Appl. Catal. B-Environ..

[33] Hu C., Lin Y., Connell J.W., Cheng H.M., Gogotsi Y., Titirici M.M., Dai L. (2019). Carbon-Based Metal-Free Catalysts for Energy Storage and Environmental Remediation. Adv. Mater..

[34] Zhang W., Low J., Long R., Xiong Y. (2020). Metal-free electrocatalysts for nitrogen reduction reaction. EnergyChem.

[35] Zhou S., Pei W., Zhao Y., Yang X., Liu N., Zhao J. (2021). Low-Dimensional Non-Metal Catalysts: Principles for Regulating P-Orbital-Dominated Reactivity. npj Comput. Mater..

[36] Xue D., Xia H., Yan W., Zhang J., Mu S. (2020). Defect Engineering on Carbon-Based Catalysts for Electrocatalytic CO_2_ Reduction. Nano-Micro Lett..

[37] Mesquita W.D., de Jesus S.R., Oliveira M.C., Ribeiro R.A.P., Santos M.R.d.C., Junior M.G., Longo E., Gurgel M.F.d.C. (2021). Barium strontium titanate-based perovskite materials from DFT Perspective: Assessing the Structural, Electronic, Vibrational, Dielectric and Energetic Properties. Theor. Chem. Acc..

[38] Cao L., Wang R., Wang D. (2015). Synthesis and Characterization of Sulfur Self-Doped g-C_3_N_4_ with Efficient Visible-Light Photocatalytic Activity. Mater. Lett..

[39] Ge L., Han C., Xiao X., Guo L., Li Y. (2013). Enhanced Visible Light Photocatalytic Hydrogen Evolution of Sulfur-Doped Polymeric g-C_3_N_4_ Photocatalysts. Mater. Res. Bull..

[40] Kresse G., Kresse G. (1999). From Ultrasoft Pseudopotentials to the Projector Augmented-Wave Method. Phys. Rev. B.

[41] Ruan L., Zhu Y., Qiu L., Lu Y. (2014). Mechanical Properties of Doped g-C_3_N_4_—A First-Principle Study. Vacuum.

[42] Kresse G., Furthmiiller J. (1996). Efficiency of Ab-Initio Total Energy Calculations for Metals And Semiconductors using a Plane-Wave Basis Set. Comp. Mater. Sci..

[43] Perdew J.P., Burke K., Ernzerhof M. (1996). Generalized Gradient Approximation Made Simple. Phys. Rev. Lett..

[44] Krukau A.V., Vydrov O.A., Izmaylov A.F., Scuseria G.E. (2006). Influence of the Exchange Screening Parameter on the Performance of Screened Hybrid Functionals. J. Chem. Phys..

[45] Heyd J., Scuseria G.E., Ernzerhof M. (2003). Hybrid Functionals Based on a Screened Coulomb Potential. J. Chem. Phys..

[46] Grimme S., Ehrlich S., Goerigk L. (2011). Effect of the Damping Function in Dispersion Corrected Density Functional Theory. J. Comput. Chem..

[47] Liu A.Y., Cohen M.L. (1989). Prediction of New Low Compressibility Solids. Science.

[48] Ding K., Wen L., Huang M., Zhang Y., Lu Y., Chen Z. (2016). How does the B,F-Monodoping and B/F-Codoping Affect the Photocatalytic Water-Splitting Performance of g-C_3_N_4_?. Phys. Chem. Chem. Phys..

[49] Azofra L.M., MacFarlane D.R., Sun C. (2016). A DFT Study of Planar vs. Corrugated Graphene-Like Carbon Nitride (g-C_3_N_4_) and its Role in the Catalytic Performance of CO_2_ Conversion. Phys. Chem. Chem. Phys..

[50] Liu G., Niu P., Sun C., Smith S.C., Chen Z., Lu G.Q.M., Cheng H.-M. (2010). Unique Electronic Structure Induced High Photoreactivity of Sulfur-Doped Graphitic C_3_N_4_. J. Am. Chem. Soc..

[51] Luo K., Chen S., Duan C. (2015). Indirect-Direct Band Gap Transition of Two-Dimensional Arsenic Layered Semiconductors—Cousins of Black Phosphorus. Sci. China Phys. Mech..

[52] Zuluaga S., Liu L.H., Shafiq N., Rupich S.M., Veyan J.F., Chabal Y.J., Thonhauser T. (2015). Structural Band-Gap Tuning in g-C_3_N_4_. Phys. Chem. Chem. Phys..

[53] Xiong W., Wang J.W., Fan W.J., Song Z.G., Tan C.S. (2020). The Theoretical Direct-Band-Gap Optical Gain of Germanium Nanowires. Sci. Rep..

[54] Lu C., Chen R., Wu X., Fan M., Liu Y., Le Z., Jiang S., Song S. (2016). Boron Doped g-C_3_N_4_ with Enhanced Photocatalytic UO_2_^2+^ Reduction Performance. Appl. Surf. Sci..

[55] Dong G., Zhang Y., Pan Q., Qiu J. (2014). A Fantastic Graphitic Carbon Nitride (g-C_3_N_4_) Material: Electronic Structure, Photocatalytic and Photoelectronic Properties. J. Photochem. Photobiol. C Photochem. Rev..

[56] Li H., Zhang Z., Liu Y., Cen W., Luo X. (2018). Functional Group Effects on the HOMO-LUMO Gap of g-C_3_N_4_. Nanomaterials.

[57] Wang K., Li Q., Liu B., Cheng B., Ho W., Yu J. (2015). Sulfur-Doped g-C_3_N_4_ with Enhanced Photocatalytic CO_2_-Reduction Performance. Appl. Catal. B-Environ..

