# Supplementary material for: Synergistic Effects of B-F/B-S and Nitrogen Vacancy Co-Doping on g-C3N4 and Photocatalytic CO2 Reduction Mechanisms: A DFT Study
Source: Molecules. 2022 Nov 6;27(21):7611. doi: 10.3390/molecules27217611 (PMC9655722; doi:10.3390/molecules27217611)
Supplement: Supplementary file 1 [file molecules-27-07611-s001.zip › molecules-1958754-supplementary.pdf]

Synergistic effects of B-F/B-S and nitrogen vacancy co-doping on g-C_3N_4 and photocatalytic CO_2 reduction mechanisms: A DFT study

Gang Fu, Xiaozhuo Song, Siwei Zhao, and Jiaxu Zhang*

School of Chemistry and Chemical Engineering

Harbin Institute of Technology

Harbin 150001, P. R. China

Author E-mail Address: zhjx@hit.edu.cn

Table S1. Formation energy (eV) of B-F-N_v and B-S-N_v co-doped g-C_3N_4.

	B-F-N_v		B-S-N_v	
	E_{doped}	E_{form}	E_{doped}	E_{form}
N_v1	-467.79	3.62	-459.07	3.25
N_v2	-468.11	3.30	-461.25	1.07
N_v3	-467.70	3.71	-458.97	3.35
N_v4	-471.96	-0.55	-459.78	2.54
N_v5	-466.05	5.35	-456.33	5.99

Table S2. The bond length parameters of intrinsic and doped g-C_3N_4.

g-C_3N_4					B-F			B-F-N_v		B-S-N_v	
bond length/Å	our work	PBE[a]	PW91[b]	Expt[b]	bond length/Å	our work	PW91[b]	bond length/Å	our work	bond length/Å	our work
a[c]	7.13	7.15	7.13	7.30	—	—	—	—	—	—	—
N1-C2	1.47	1.47	1.47	—	B-N1	1.51	1.51	B-N1	1.62	B-N1	1.53
C2-N3	1.33	1.34	1.34	—	B-N2	1.49	1.47	B-N2	1.54	B-S	1.98
N3-C4	1.33	1.33	1.33	—	N2-C3	1.38	1.37	N2-C3	1.19	S-C2	1.76
C4-N5	1.39	1.39	1.40	—	B-N6	1.40	1.41	B-N5	1.55	C2-N3	1.39
—	—	—	—	—	F-N2	1.41	1.41	B-F	1.40	B-N5	1.38

[a] The parameters are from ref. [28]. [b] The parameters are from ref. [48]. [c] a represents the unit cell parameter.

Table S3. The band gap energy (eV) for the intrinsic and doped g-C_3N_4.

	E_g/eV			
	PBE	HSE06		Expt
g-C_3N_4	1.21	2.77	2.68[a]	2.73[b], 2.77[c]
B-F	1.83	3.06	2.94[a]	2.86[c]
B-F-N_v	1.19	2.67	—	
B-S-N_v	1.16	—	—	

[a] The results for g-C_3N_4 and B-F are from ref. [31]. [b] The value is from ref. [50]. [c] The results for g-C_3N_4 and B-F are from ref. [28].

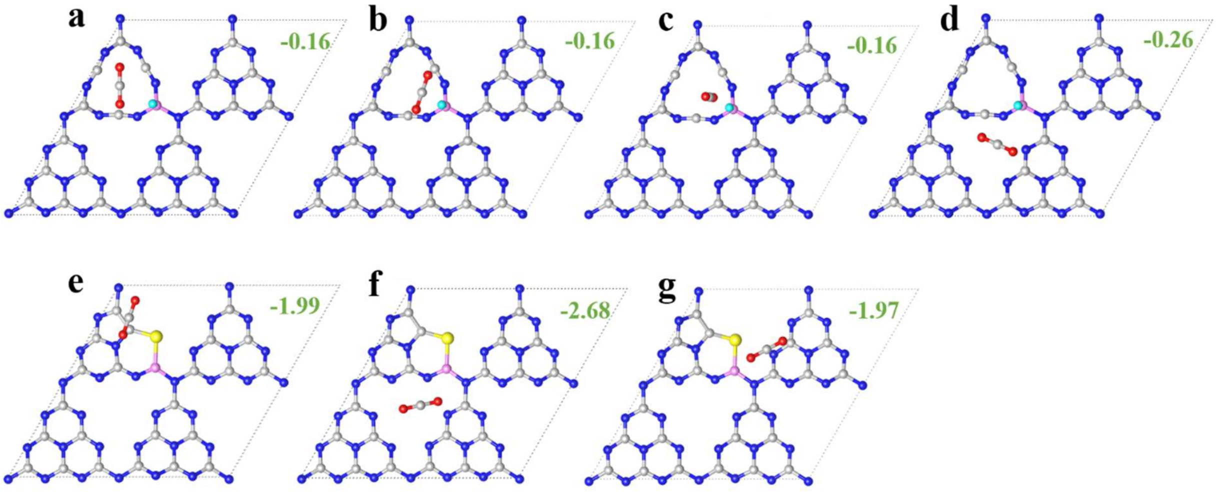

Figure S1. The possible adsorption sites for CO_2 on B-F-N_v (a-d) and B-S-N_v (e-g), and corresponding adsorption energy (eV).

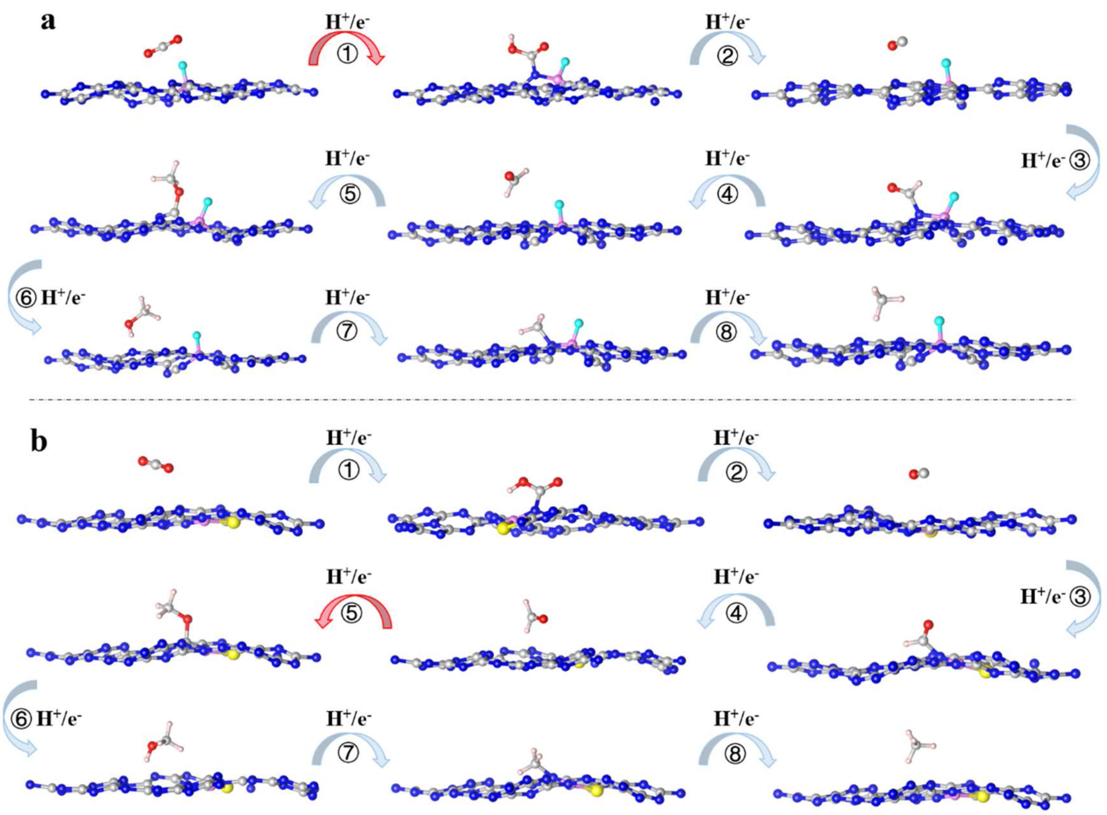

Figure S2. The reaction pathways for B-F-N_v (a) and B-S-N_v (b).

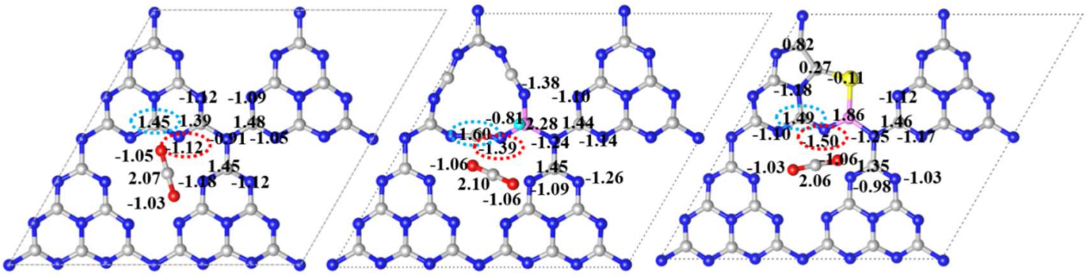

Figure S3. The bader charge (unit e) of g-C_3N_4 (left), B-F-N_v (middle), and B-S-N_v (right). Negative and positive values mean gaining and losing electrons. The elements are colored in pink for B, gray for C, blue for N, red for O, cyan for F, and yellow for S.

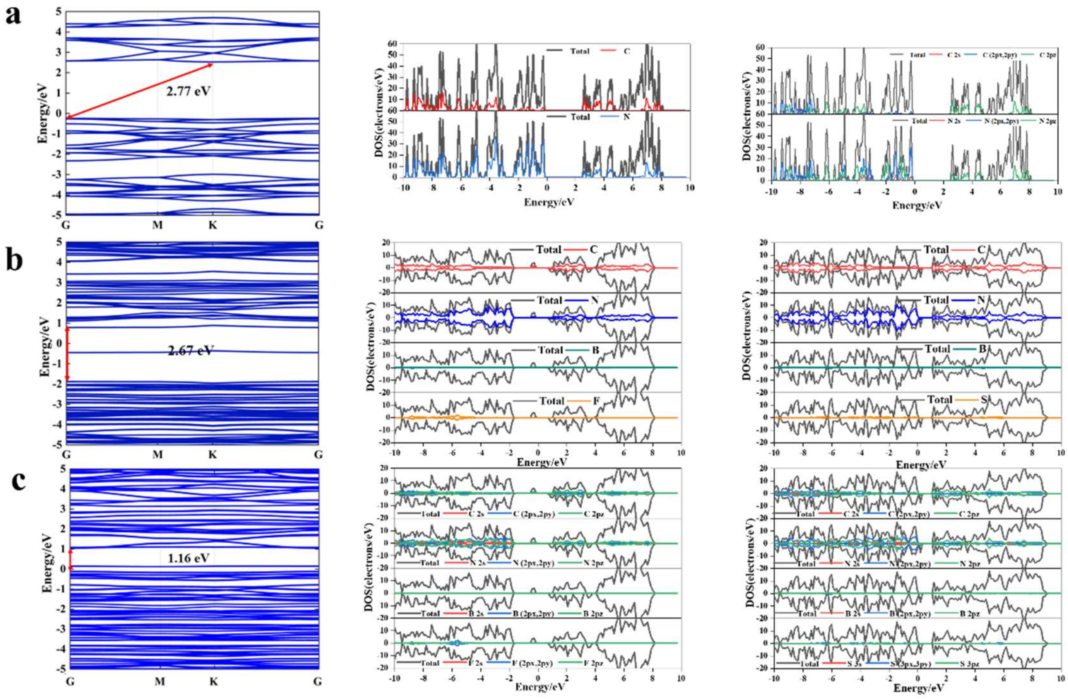

Figure S4. Calculated band structure (left), density of states (middle), and partial density of states (right) diagrams of g-C_3N_4 (a), B-F-N_v (b) and B-S-N_v (c), the energy range is from -10 eV to 10 eV.
